# Non-infectious Inflammatory Lesions of the Sinonasal Tract

**DOI:** 10.1007/s12105-016-0689-6

**Published:** 2016-02-01

**Authors:** Timothy R. Helliwell

**Affiliations:** University of Liverpool, Liverpool, UK

**Keywords:** Nose and paranasal sinuses, Rhinosinusitis, Granulomatous inflammation, Review

## Abstract

This review covers the histopathology and pathogenesis of non-infectious inflammatory diseases of the sinonasal tract, in particular, sarcoidosis, granulomatous vasculitides Wegener, Churg–Strauss), relapsing polychondritis, eosinophilic angiocentric fibrosis, chronic rhinosinusitis and nasal perforations. Molecular associations and mechanisms are emphasised to assist pathologists to put their observations into the context of clinical, genetic and environmental influences on patients’ diseases.

## Introduction

Non-infectious inflammatory diseases of the sinonasal tract include a spectrum of common and uncommon conditions, the aetiology and pathogenesis involving genetic, immunological and environmental influences. The histopathological features are well described but often elusive in biopsy material. This review focusses on recent advances in the understanding of the molecular and cellular pathophysiology of these diseases to help pathologists put their observations into the clinical context.

## Sarcoidosis

Sinonasal involvement in sarcoidosis is usually part of multisystem disease, but patients may present with chronic rhinosinusitis, nasal obstruction and nasal crusting. The clinico-pathologic features required for diagnosis [1] are:

- Mucoperiosteal thickening or opacification of a sinus in imaging.
- Histopathologic demonstration of non-caseating granulomata in the upper respiratory tract. Stains for fungi and mycobacteria should be negative, and there should be no vasculitis or cholesterol crystals.
- Negative serology for syphilis and anti-neutrophil cytoplasmic antibody.
- Exclusion of other disease processes associated with granulomatous inflammation, including tuberculosis, Wegener’s granulomatosis, and fungal infection.

Four clinical patterns of disease are identified: mucosal hypertrophy, mucosal atrophy, nasal destruction and nasal enlargement [2]. Histological and other laboratory investigations may be required to resolve some of the clinical differential diagnoses, although biopsies are often non-specific (Table 1).

**Table 1:** Clinical subtypes of sarcoidosis with their corresponding differential diagnoses

Clinical subtype of sinonasal sarcoidosis	Differential diagnosis	Diagnostic clinical and histological features^a^
Atrophic	Wegener’s granulomatosis	Granulomatous vasculitis
	Cicatricial pemphigus	Skin and other mucosal involvement
	Linear IgA dermatosis	Positive IgA immunofluorescence
	Rhinoscleroma	Characteristic histology
	Atrophic rhinitis	Extreme crusting and nasal fetor
Hypertrophic	Allergic rhinitis	
	Fungal or bacterial rhinosinusitis	Specific organisms
	Churg–Strauss	Eosinophilic vasculitis
Destructive	Wegener’s granulomatosis	Granulomatous vasculitis
	NK/T cell lymphoma	Atypical lymphoid infiltrate
	Cocaine abuse	Clinical history, p-ANCA
Nasal enlargement	Massive polyposis	
	Fibrous dysplasia	Imaging studies

^a^These diagnostic clinical and histological features may not be present in all patients

### Immunology and Genetic Susceptibility

Sarcoidosis involves a dysregulated immune response to a range of environmental stimuli in genetically susceptible people. Possible environmental factors include fungi, inorganic particles and insecticides [3]. Sarcoidosis is 2.5 times more common in the siblings and parents of affected patients and is three times more common and more severe in African Americans than European Americans [3]. Genome wide surveys have demonstrated linkage with the NOTCH4 gene in African Americans and show consistent linkage with polymorphisms of the HLA-DRA, HLA-DRB (HLA-B8/DR3 haplotype and HLADRB*1101) and HLA-DRC genes [3–5]. There is a significant overlap of the molecular pathology of sarcoidosis and that of other immunologically mediated inflammatory disorders, with a prominent role of the drug-targetable IL23/T_H_17-signaling pathway [5].

### Histopathology

**Fig. 1:**
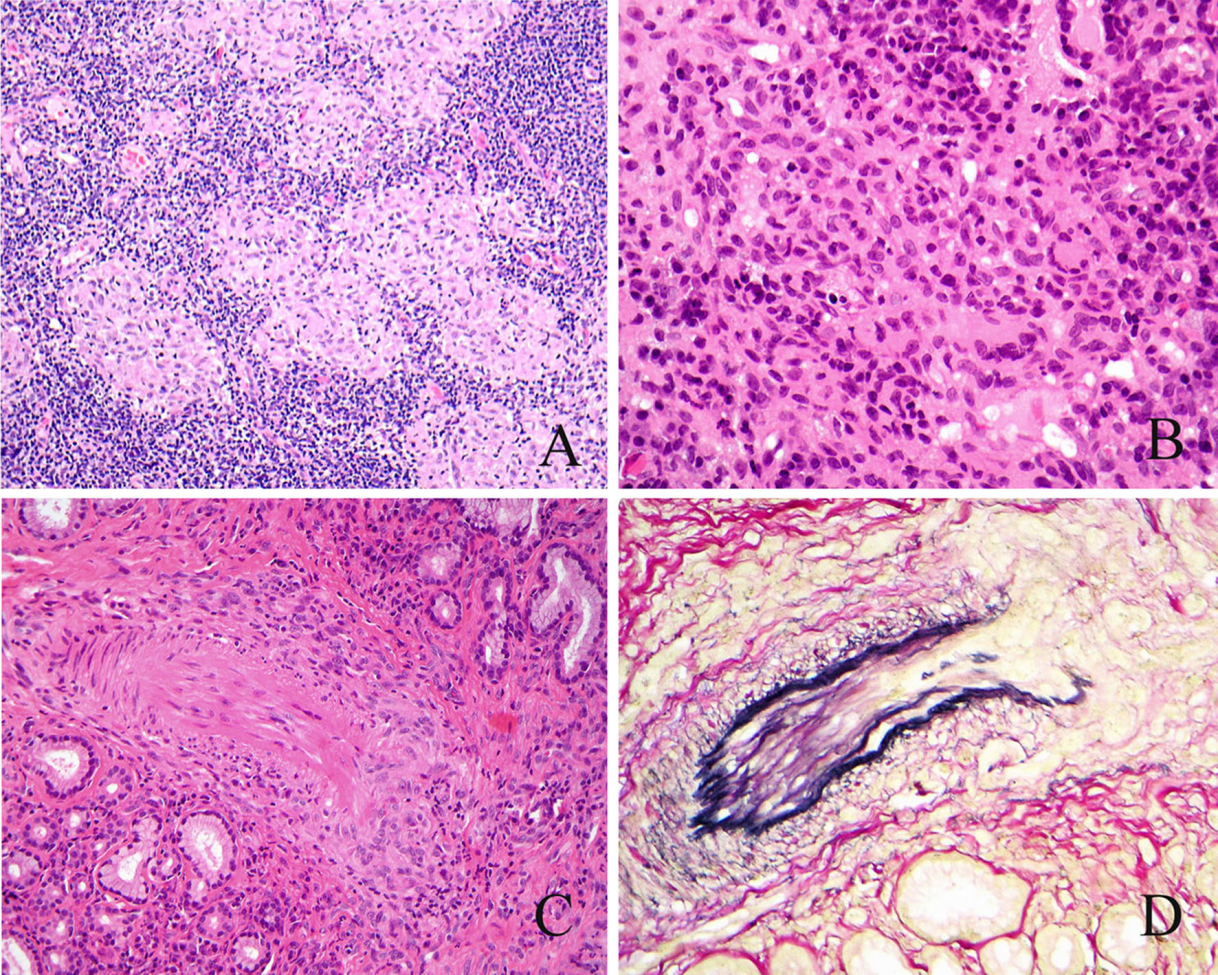
**a** Nasal sarcoidosis. Irregular, pale epithelioid cell granulomas are surrounded by small lymphocytes. **b** Granulomatosis with polyangiitis (Wegener). Poorly formed epithelioid and multinucleate cell granulomas are associated with small lymphocytes. **c** Granulomatosis with polyangiitis (Wegener). Disruption of the wall of this small mucosal vessel is seen on H&E staining and **d** on staining for elastic tissue (EVG)

The diagnostic histological features of mucosal biopsies comprise non-caseating epithelioid cell granulomas associated with an infiltrate of small T lymphocytes (Fig. 1a). Multinucleate cells are infrequent and there is rarely more than minimal necrosis. With time, increasing fibrosis is associated with the granulomatous response.

## Granulomatosis with Polyangiitis (GPA, Wegener’s Granulomatosis)

### Terminology

Small vessel vasculitides are grouped into immune complex vasculitis and those vasculitis syndromes associated with anti-neutrophil cytoplasmic antibodies (ANCA) in which there is little evidence of immune complex deposition [6]. ANCA-associated necrotising vasculitides (AAV) include microscopic polyangiitis, granulomatosis with polyangiitis (GPA, Wegener) and eosinophilic granulomatosis with polyangiitis (EGPA, Churg–Strauss).

### Aetiology and Pathogenesis

The aetiology of GPA is unknown but is likely to involve a range of environmental factors, including micro-organisms, in genetically susceptible individuals. GPA is associated in 80 % patients with c-ANCA antibodies to proteinase 3, and in 20 % patients with antibodies to myeloperoxidase. Proteinase 3 is found in the azurophilic granules of neutrophils and is expressed on the membranes of secretory vacuoles and on the surface of resting neutrophils; the extent of this constitutive expression is very variable (0–100 % neutrophils) and is genetically determined [7]. The characteristic cytokine profile in GPA indicates a T_H_1/T_H_17 response with the production of cytokines such as TNF-α and IFN-γ which are probably linked to the granulomatous response. HLA-1 involvement is also likely, but the strongest HLA associations are with HLA-DR4 and HLA-DPB1 [4].

### Clinical Features

GPA affects males and females equally, with most patients presenting over the age of 45 years with sinonasal symptoms of obstruction, bleeding or crusting associated with pulmonary infiltrates and renal impairment [4, 8]. Even in the absence of active disease, the loss of the normal mucosal functions may lead to dry mucosa and repeated infections. Chronic disease may cause meatal obstruction, sinus neo-ossification, septal destruction and the collapse of the nasal cartilages. Oral ulceration, gingivitis and subglottic stenosis are seen in a minority of patients. Treatment with corticosteroids and immunosuppressive agents has greatly reduced the mortality of GPA, with 10-year survival rates now exceeding 80 % [8]. Treatment associated morbidity is a challenge and biological response modifiers targeting proteinase 3 and cathepsin C may become more important.

### Histopathology

Most mucosal biopsies show non-specific active chronic inflammation. The characteristic features, when present, are irregular areas of necrosis associated with multinucleate cells and poorly formed epithelioid cell granulomas associated with T lymphocytes and vasculitis Fig. 1b). Vasculitis affects small and medium-sized vessels and ranges from leucocytoclastic vasculitis to classical vasculitis with fibrinoid necrosis [9] (Fig. 1c, d). Biopsies should be used to exclude possible differential diagnoses including infectious granulomas, sarcoidosis, and mucosal malignancies, particularly NK/T cell lymphoma.

## Eosinophilic Granulomatosis with Polyangiitis (EGPA, Churg–Strauss Syndrome)

**Fig. 2:**
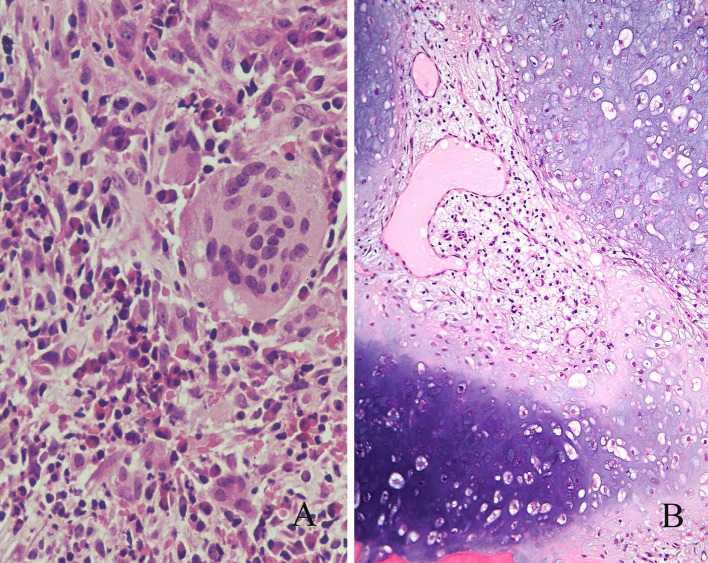
**a** Eosinophilic granulomatosis with polyangiitis (Churg–Strauss). The typical eosinophil-rich granulomatous infiltrate is present in this nasal biopsy. **b** Relapsing polychondritis. Cartilage erosion by mononuclear cells and vascular granulation tissue

EGPA is a rare systemic vasculitis that was first described in patients diagnosed as polyarteritis nodosa with necrotising vasculitis, but who showed tissue and blood eosinophilia and extravascular granulomas [10]. Patients have symptoms of allergic rhinitis, polyposis and sinusitis and a history of asthma [11]. Clinically significant disease often affects the lungs, skin and kidneys, although severe renal disease is uncommon. Consensus recommendations for evaluation and management have been published [12]. Diagnosing EGPA implies biopsy-proven vasculitis or a strong clinical surrogate, but either can be difficult to obtain. Within the clinical contexts of asthma with eosinophilia, asthma with systemic manifestations or with extrapulmonary disease, a biopsy demonstrating small- or medium-sized-vessel vasculitis or pauci-immune crescentic glomerulonephritis, strongly supports a diagnosis of EGPA. Mucosal biopsies typically show an eosinophil-rich inflammatory infiltrate and may also show vasculitis and granulomatous inflammation (Fig. 2a) [11].

### Immunopathology

A perinuclear immunofluorescent pattern detecting ANCA to myeloperoxidase (MPO) is the most common EGPA ANCA-positivity finding. Anti-proteinase-3 (PR3) ANCA antibodies have been reported in EGPA, but are unusual. ANCA-positive patients are more likely to have a vasculitic phenotype with glomerulonephritis and mononeuritis multiplex, while the prognosis of ANCA-negative patients is poorer, possibly because of their higher frequency of cardiomyopathy [12]. The inflammatory response in EGPA is predominant related to T_H_2 lymphocytes. Associated HLA alleles are HLA-DRB1, HLA-DRB3 and HLA-DRB3 genes but not with HLA-DPB140 (as in GPA) [4].

## Relapsing Polychondritis

Relapsing polychondritis is a rare disease of young and middle aged adults with recurrent painful episodes of acute inflammation that destroy the nasal and aural cartilages. Tracheal chondritis leads to airway obstruction and pneumonia. Other manifestations include migratory arthritis, ocular inflammation, cardiac and neurological disease [13]. About one-third of patients have other autoimmune diseases and there is overlap with Behcet’s disease—the so-called MAGIC syndrome of mouth and genital ulcers with inflamed cartilage [14]. Relapsing polychondritis is a T_H_1 mediated disease and, although the target antigens are not known, human and experimental studies suggest that collagen type II and the cartilage matrix protein matrilin-1 are likely candidates [13]. Biopsies of involved cartilage shows loss of mucopolysaccharide matrix, disruption of elastic fibres and microcysts, with erosion by CD4+ T lymphocytes (Fig. 2b). Neutrophils may be present in the early stages and there is usually perichondrial granulation tissue and fibrosis [13, 15].

## Eosinophilic Angiocentric Fibrosis

**Fig. 3:**
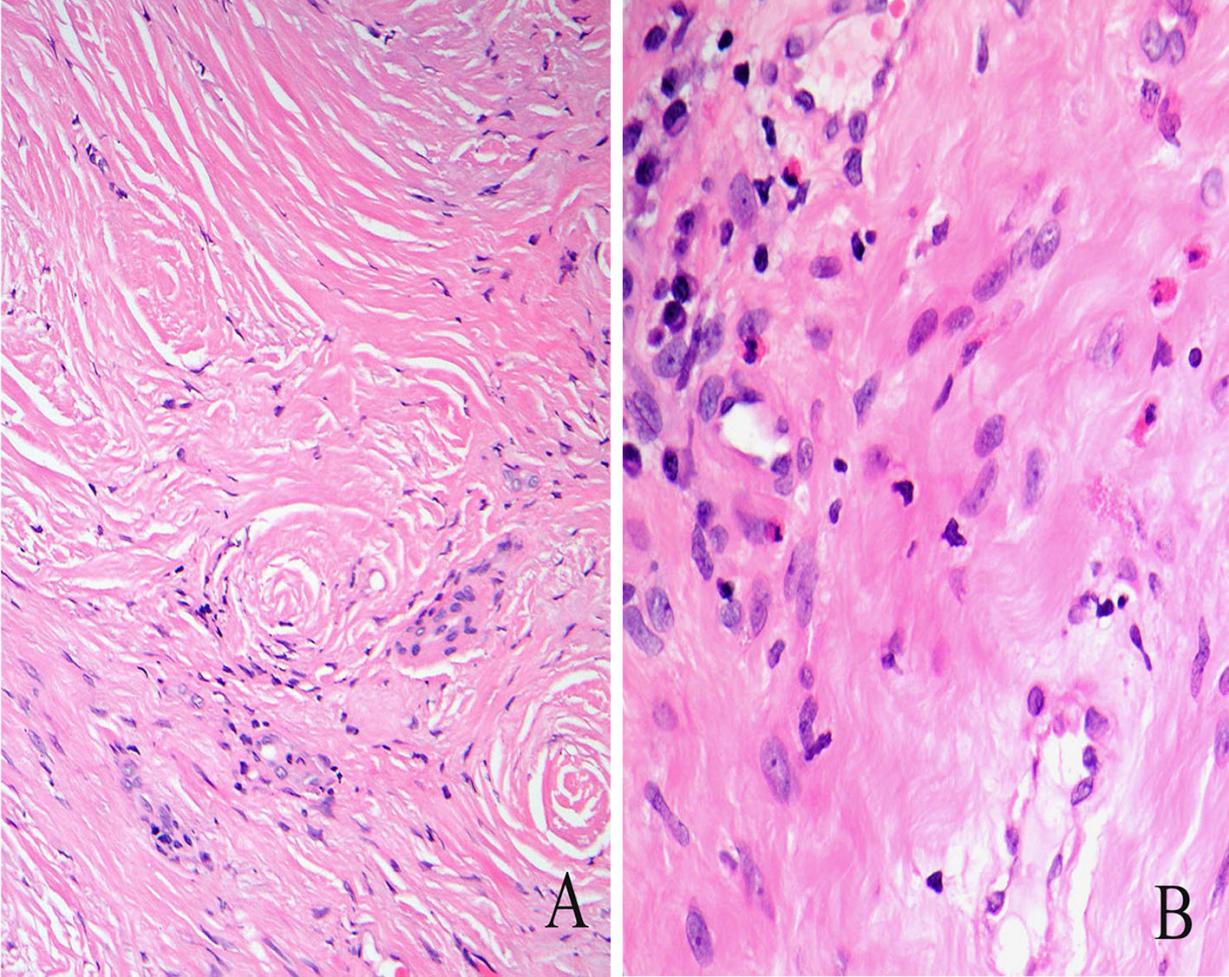
Eosinophilic angiocentric fibrosis. Late stage disease with **a** dense perivascular fibrosis and **b** a scanty infiltrate of plasma cells and eosinophils

Eosinophilic angiocentric fibrosis is an uncommon, idiopathic inflammatory condition of the nose affecting middle-aged adults with progressive fibrosis leading to nasal obstruction. EAF is considered to be one of the IgG4 related diseases which are typified by storiform fibrosis, obliterative phlebitis, many IgG4-positive plasma cells, a ratio of IgG4:total IgG plasma cells of >40 % and raised serum IgG4 [16, 17]. In the early stages, there is an eosinophil-rich inflammatory exudate around vessels. In later stages the inflammatory infiltrate is more patchy and mixed (eosinophils, neutrophils, lymphocytes and plasma cells) and progresses to onion-skin perivascular fibrosis (Fig. 3). The clinical and imaging features are non-specific and the diagnosis is made histologically [18].

## Chronic Rhinosinusitis

Chronic rhinosinusitis (CRS) is a common disease, defined clinically as inflammation of the sinonasal mucosa leading to symptoms of nasal obstruction and discharge for more than 3 months. Many factors are implicated in chronic rhinosinusitis, including genetic, immunological and environmental factors [19]. Although antimicrobial treatment has little effect on the symptoms of CRS, bacteria and fungi are implicated in the pathogenesis of CRS through their involvement in the biofilms that form on the surface of the sinonasal mucosa. Biofilms are collections of micro-organisms that grow in a self-produced matrix, conditions that lead to modifications in gene expression. Sinonasal biofilms contain Staphylococcus aureus, Streptococcus pneumonia and Pseudomonas aeruginosa, sometimes associated with fungi such as *Aspergillus* sp. [4].

The inherited disorders associated with CRS are cystic fibrosis and primary ciliary dyskinesia. Cystic fibrosis is caused by a mutation in the cystic fibrosis transmembrane conductance regulator gene (7q31.2) leading to a defect in chloride transport and thick mucin. As the life expectancy of patients with cystic fibrosis has increased in recent years, most patients develop CRS and nasal polyposis. Histologically, polyps in patients with CF are similar to those in other forms of CRS and there is a similar pattern of gene expression in mucosal glands, with the exception of increased expression in CF patients of the orthodenticle homeobox 2 gene, OTX2 [4, 20].

Primary ciliary dyskinesia syndrome is a group of conditions in which structural and functional abnormalities of cilia lead to CRS with polyps and recurrent infections. About 50 % of patients have Kartagener syndrome with CRS, situs inversus and bronchiectasis [21].

Inhaled water-soluble irritants such as formaldehyde, ammonia and sulphur dioxide readily dissolve in the surface mucus and may cause changes in the structure and function of the mucosa leading to olfactory dysfunction, sensory irritation and rhinosinusitis. Occupational rhinitis is associated with a wide range of chemicals and may be non-allergic or allergic in pathogenesis [22].

### Immunopathology

For clinical purposes, it is usual to consider allergic rhinitis, non-allergic rhinitis and chronic rhinosinusitis as separate but overlapping conditions, with or without polyposis (Table 2). Allergic rhinitis is an IgE-mediated response to inhaled allergens, in which activation of the T_H_2 pathway stimulates IL3, IL4, IL5 and IL13 production leading to local and systemic IgE synthesis. In tropical urban environments, exposure to dust mites is prevalent while in other parts of the world multiple allergens are implicated [23, 24].

**Table 2:** Clinico-pathological subtypes of rhinitis

Allergic rhinitis
Seasonal rhinitis—tree pollen (spring), grass pollen (summer), weed pollen (late summer) and fungal spores (autumn and winter)
Perennial rhinitis—allergens found in the faeces of the house dust mite.
Non-allergic rhinitis with eosinophilia (NARES)
Non-allergic, non-eosinophilic rhinitis

Non-infectious non-allergic rhinitis may be influenced by allergic conditions, medications and hormonal factors, with a subgroup of patients showing general nasal hyper-reactivity to inhaled irritants.

Non-allergic rhinitis with eosinophilia syndrome (NARES) is most prevalent in middle-aged adults who have symptoms of rhinitis with polyposis in the absence of atopy. The nasal mucosa and fluid contains eosinophils [25]. IgE levels are not elevated and anosmia is a prominent feature. The pathophysiology is poorly understood. Non-allergic rhinitis with a predominance of neutrophils and/or mast cells has been suggested by some authors to have different clinical features and treatment [26].

Non allergic non-eosinophilic rhinitis is most prevalent in middle aged and elderly adults and is related to mechanical obstruction to drainage and secondary bacterial infection. Symptoms are triggered by changes in temperature, light intensity and emotion, probably due to hyper-reactivity of the autonomic nervous system leading to the release of vasoactive neurotransmitters.

### Pathogenesis

The pathogenesis of the inflammatory changes in CRS is complex [27, 28]. Activated mast cells and fibroblasts produce proinflammatory cytokines (Il-1, Il-6, interferon-γ and TNF-α) which attract and activate neutrophils and eosinophils [29]. Lymphocyte subpopulations differ between patients without polyposis (T_H_1 predominant with high levels of interferon-γ) and those with polyposis (T_H_2 predominant with high IL-5 and IgE levels) [27]. Mucosal tissue remodelling is manifest as breakdown of the extracellular matrix by proteases (MMP-9) leading to pseudocysts, basement membrane thickening by fibronectin and types I, III and V collagens, and mucosal gland hyperplasia [29]. TGF-β produced by eosinophils stimulates fibroblasts, as well as upregulating VEGF expression which induces angiogenesis and oedema [28]. Tissue eosinophilia is found in up to 90 % of mucosal biopsies but the role of eosinophils is uncertain, being found in both allergy and NARES.

### Histopathology

**Fig. 4:**
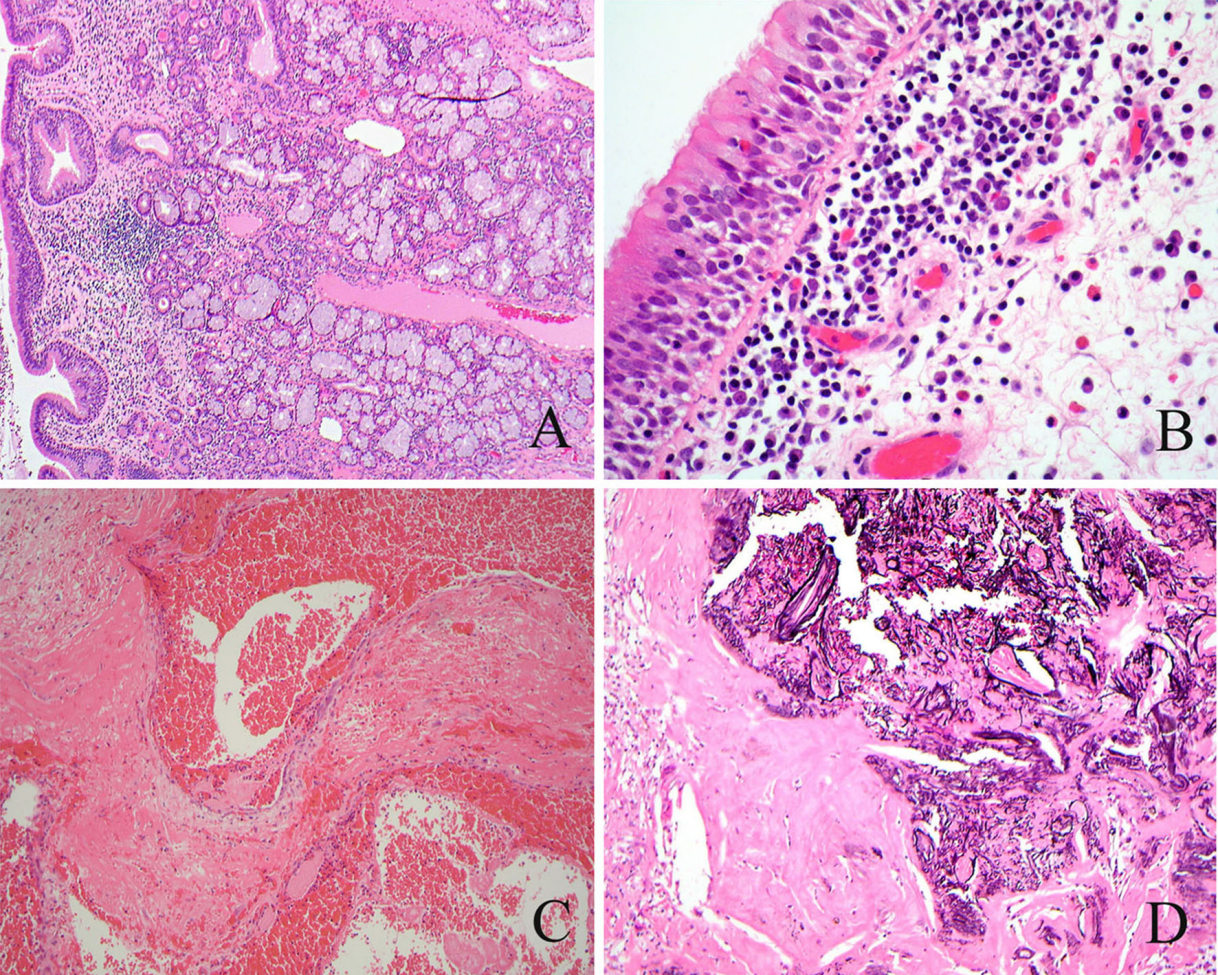
**a** Chronic rhinosinusitis. Mucosal gland hyperplasia. **b** Chronic rhinosinusitis. Inflammatory infiltrate rich in eosinophils. **c** Inflammatory nasal polyp with haemorrhage and abundant fibrin. **d** Inflammatory nasal polyp with partly calcified cholesterol granuloma

Nasal cytology is of limited value in the assessment of CRS, but may demonstrate eosinophils. Nasal biopsies are useful for the exclusion of granulomatous disorders, vasculitis and, for unilateral disease, neoplasia. Histologically, chronic rhinosinusitis is characterised by mucosal thickening with goblet cell hyperplasia (Fig. 4a), submucosal fibrosis and a non-specific infiltrate of lymphocytes and plasma cells, with or without eosinophils.

Inflammatory polyps are smooth-surfaced, pink or pale brown masses up to 3-4 cm in diameter, attached to the mucosa of the lateral nasal cavity close to the sinus ostia. Polyps are covered by ciliated respiratory epithelium and may show immature squamous metaplasia and ulceration. The epithelial basement membrane is thickened and the stroma shows marked oedema and myxoid change with pseudocystic degeneration and variable numbers of blood vessels and myofibroblasts. The intensity and type of inflammatory infiltrate varies markedly both within and between polyps; eosinophils, lymphocytes and plasma cells are present and eosinophils often predominate (Fig. 4b). Polyps from patients with asthma show more basement membrane thickening, goblet cell hyperplasia and eosinophilic infiltration than polyps from patients without asthma [30], but this is not of practical importance.

Secondary changes that may create problems in diagnosis include ulceration and infection with a predominantly neutrophilic infiltrate, fibrosis, infarction, granulation tissue, the deposition of dense fibrinous material resembling amyloid (Fig. 4c), cartilaginous and osseous metaplasia, and cholesterol granuloma formation with calcification (Fig. 4d).

## Nasal Septal Perforation

Nasal septal perforation may be caused by local trauma (‘nose-pickers’ perforation), inhalation of toxic substances such as cocaine or intranasal steroid and decongestant sprays, infective and non-infective granulomatous diseases and nasal malignancies [31]. Occupational exposure to corrosive chemicals such as strong acids and alkalis, chromates used in the electroplating industry, nickel, arsenic and copper may lead to perforation [32]. Biopsies are taken from the mucosal edge of a perforation to exclude specific pathologies, and usually show non-specific acute inflammatory debris, inflamed granulation tissue and reactive changes in the adjacent epithelium.

### Cocaine*-*Induced Midline Destructive Lesions (CIMDL)

CIMDL most probably arise from the intense vasoconstriction and extensive destruction of the cartilaginous tissues of the nose following inhalation of cocaine. The main clinical differential diagnosis is with GPA, and there is substantial overlap in histopathology and autoantibody profiles. Biopsies tend to show non-specific chronic inflammation and necrosis but may include leucocytoclastic vasculitis and a granulomatous reaction to foreign material which should be distinguished from the more deeply situated granulomas with necrosis sometimes seen in GPA [33]. CIMDL is associated with c-ANCA and p-ANCA directed against neutrophil elastase [33].
